# Nigeria sodium study 2023 policy meeting on dietary sodium reduction in Nigeria

**DOI:** 10.1186/s12919-024-00303-3

**Published:** 2024-09-18

**Authors:** Adedayo E. Ojo, Vanessa O. Alfa, Mark D. Huffman, Dike B. Ojji

**Affiliations:** 1 Cardiovascular Research Unit, University of Abuja and University of Abuja Teaching Hospital, Gwagwalada, 902101 Abuja, Nigeria; 2 Julius Global Health, Julius Center for Health Sciences and Primary Care, University Medical Center, Utrecht, 3584 CG The Netherlands; 3 Cardiovascular Division and Global Health Center, Washington University, St. Louis, MO 63110 USA; 4 The George Institute for Global Health, University of New South Wales, Sydney, NSW 2052 Australia; 5 Department of Preventive Medicine, Feinberg School of Medicine, Northwestern University, Chicago, IL 60611 USA; 6 Department of Medicine, Faculty of Clinical Sciences, University of Abuja, Gwagwalada, Abuja, 902101 Nigeria

**Keywords:** Sodium intake, Hypertension, Cardiovascular disease, Sodium reduction policies, Nigeria

## Abstract

**Background:**

In line with the WHO Global Action Plan’s objective to achieve a 30% relative reduction in the mean population intake of sodium by 2025, the Nigeria Sodium Study (NaSS) team, which aimed to evaluate the implementation and scale-up of national sodium reduction programme, hosted a policy meeting May 22, 2023, in Abuja, the Federal Capital Territory of Nigeria. The aim was to deliberate on strategies for translating data on salt levels in food and dietary patterns, intending to strengthen sodium policies in Nigeria, with the ultimate goal of producing evidence-based information that can effectively guide strategies and policies for sodium reduction.

**Methods:**

Policymakers from federal, state, and local government levels attended, as well as representatives from national and international universities and non-governmental organizations. Topics presented and discussed included retail survey data from the NaSS to inform front-of-package labeling, salt targets for packaged food, and best practices for supporting stakeholders in implementing best-practice evidence-informed policymaking.

**Results:**

The meeting brought together 72 participants from 38 organizations, including government ministries and agencies (*n* = 21), international and non-governmental organizations (*n* = 6), and international health organizations and institutes (*n* = 2). Participants took decisive policy actions, including stringent national-level food system monitoring by relevant government agencies, implementing front-of-package labeling for healthier choices, establishing mandatory sodium limits for both packaged and unpackaged foods and school meals, launching diverse sector-wide educational campaigns to reduce salt use, conducting mass mobilization campaigns for awareness, and advocating for salt reduction in fast food outlets. Salt substitutes were also recognized as integral to the comprehensive sodium reduction approach.

**Conclusion:**

To advance policy action, stakeholders should prioritize establishing robust monitoring systems, engage in public awareness campaigns, and collaborate with international organizations for insights. Exploring partnerships, addressing funding challenges, and implementing innovative strategies like low-sodium substitutes are crucial steps toward effective sodium reduction policies in Nigeria.

## Introduction

### Sodium intake, hypertension and Cardiovascular Disease (CVD)

Given the significant impact of hypertension on public health in Nigeria, with an estimated prevalence among adults ranging from 25 to 40%, there is a pressing need to address the contributing factors, particularly unhealthy dietary habits [1, 2]. Cardiovascular diseases, such as coronary artery disease, stroke, and heart failure, not only represent major sources of morbidity and mortality in Nigeria but also result in substantial healthcare expenses at the individual and household levels [3–5]. Recognizing the intricate link between excessive dietary sodium consumption, elevated blood pressure, and cardiac health, it is imperative for Nigeria to consider additional sodium reduction policies implemented successfully in other countries. By adopting proactive measures, such as reducing sodium content in processed foods**,** implementing clear food labeling**,** promoting consumer education**,** and developing national public health programs**,** as recommended by the World Health Organization (WHO), Nigeria can alleviate the burden of hypertension and cardiovascular diseases, enhancing overall public health outcomes [6].

Nigeria has taken a significant step in mitigating excess dietary sodium in the population by introducing the National Multi-Sectoral Action Plan (NMSAP) for the Prevention and Control of Non-Communicable Diseases in 2019. This comprehensive plan incorporates evidence-based policies aligned with the WHO SHAKE technical package. The SHAKE technical package serves as a foundational framework, providing scientifically grounded guidelines and strategies to address the challenges associated with sodium intake and promote overall health [7]. The national sodium reduction programme in Nigeria encompasses a multifaceted approach targeting four key policies. First, it involves the establishment and enforcement of specific limits on sodium content in packaged foods, contributing to an overall reduction in sodium intake across the population. Additionally, the program incorporates extensive mass media campaigns aimed at fostering awareness and educating the public on the advantages of healthy eating, with a specific focus on minimizing sodium consumption. Furthermore, there are regulations on food and beverage for advertisement, particularly when directed at children and adolescents, to mitigate the impact of such promotions on dietary choices. Lastly, the sodium reduction initiative integrates health education programs into school settings, intending to instil healthy habits early on by educating students about the importance of making well-informed dietary choices and the potential consequences of excessive sodium intake. The aim NaSS Policy Meeting on Dietary Sodium Reduction in Nigeria was to deliberate on strategies for translating data on salt levels in food and dietary patterns, intending to strengthen sodium policies in Nigeria, with the ultimate goal of producing evidence-based information that can effectively guide strategies and policies for sodium reduction.

## Methods

### Invitations and participants

Invitations were extended to policymakers from federal, state, and local government levels, along with representatives from national and international universities, non-governmental organizations, and various sectors involved in food research, innovation, regulatory functions, legislative functions, public health campaigns, and healthcare professionals in Nigeria. Participants were selected based on their roles in contributing to or influencing sodium policies.

### Consent processes

Prior to the meeting, participants received official invitation letters outlining the purpose and details of the event. These letters served as comprehensive documents elucidating the overarching purpose, intricate details, and significance of the forthcoming event. The invitations were designed to ensure clarity regarding the subject matter and to provide participants with a thorough understanding of the agenda, emphasizing the relevance of their contributions to the discussions. It was noted that attendance was not mandatory, and participants could withdraw at any point without consequences. This approach was instrumental in ensuring that the insights and contributions made during the meeting were driven by participants’ genuine interest and commitment to the objectives of the meeting.

### Discussion format

The meeting adopted an open discussion format, where participants actively engaged in dialogue. Topics presented included retail survey data, front-of-package labeling, salt targets, and best practices. The open discussion covered unintended consequences of dietary policies, iodine supplements, the food industry’s role, and strategies to reduce sodium intake (Table 2).

### Policymaker meeting on national sodium reduction

**Fig. 1:**
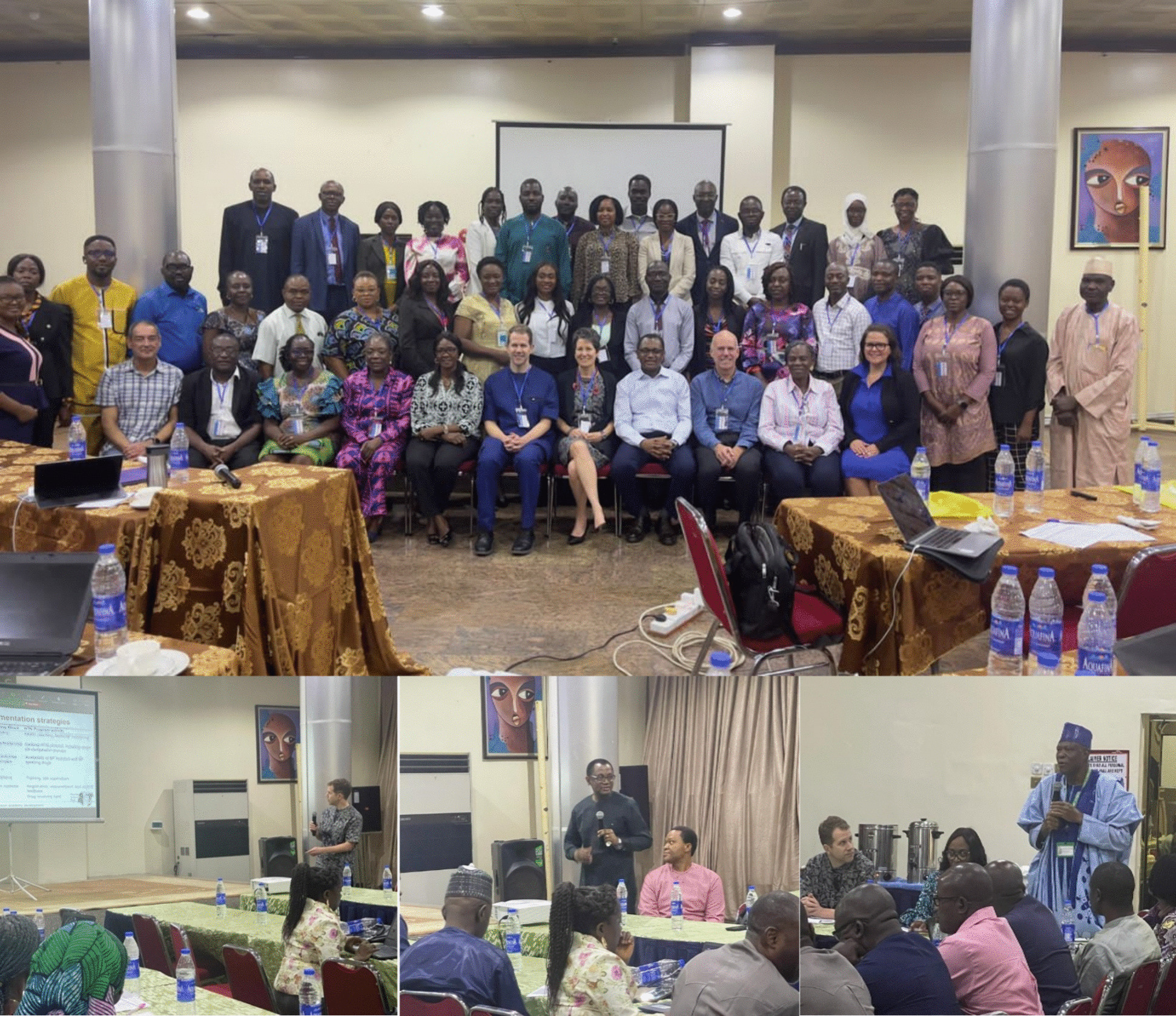
Photograph displaying the collective portrait, two co-principal investigators of the Nigeria Sodium Study (left, middle), and Vice Chancellor of the University of Abuja (right)

The organization of this meeting seamlessly aligned with the primary objectives of both the National Multi-Sectoral Action Plan (NMSAP) and the World Health Organization’s (WHO) sodium reduction initiatives. The NaSS team, composed of researchers from esteemed institutions such as the University of Abuja Teaching Hospital, Washington University in St Louis, Northwestern University, and The George Institute for Global Health, actively evaluates the implementation and scale-up of the national sodium reduction program (Fig. 1). This comprehensive assessment, supported by funding from the National Heart, Lung, and Blood Institute (NHLBI), aims to gauge the implementation, effectiveness and impact of the sodium reduction initiatives outlined in the NMSAP. The central focus of the NaSS team is to contribute evidence-based insights for informing and guiding strategies and policies for sodium reduction in Nigeria. The meeting aimed to evaluate existing and identify new strategies to reduce excess dietary sodium intake in Nigeria, with the objective of lessening the burden of diet-related cardiovascular diseases. Policymakers from federal, state, and local government levels, along with representatives from national and international universities, non-governmental and inter-governmental organizations, as well as individuals involved in food research and innovation, regulatory and legislative functions, public health campaigns, and healthcare professionals in Nigeria, attended the meeting.

Acknowledging the large and growing burden of diet-related CVDs, participants discussed policies related to food system surveillance, industry collaboration, labeling standards, knowledge dissemination, and creating a conducive environment for dietary sodium policy implementation (Tables 1 and 2):

**Table 1:** Agenda: Nigeria sodium study 2023 policy meeting—advancing strategies for dietary sodium reduction

S/N	Item
**1.**	Welcome and self-introductions: Dike Ojji/All
**2.**	Objective of the meeting:
**3.**	NASS Program updates Mark Huffman
**4.**	Sodium reduction strategies and principles: Joy Amafa
**5.**	Salt targets, Sat target policies and best practices: Nicole Ide
**6.**	Supporting stakeholders in evidence-informed policy for dietary sodium reduction: Bruce Neil and Fraiser Tailor
**7.**	Discussion and Feedback: All

**Table 2:** Points for discussion raised

1. How can the data generated from this study be translated into policies? 2. How can mandatory salt limits be established in Nigeria from this data? 3. What are the other ways academics can contribute to these processes?	

In a NASS presentation, Dike Ojji expressed gratitude for the UH3/UG3 award from the NHLBI, emphasizing the focus on improving Nigerian food consumption. The session aimed to review progress on existing strategies and brainstorm strategies, build on existing and forge new partnerships, and utilize study data for evidence-based policymaking. Ojji outlined the NaSS project, discussed the 2019 National Multisectoral Action Plan, and highlighted the upcoming STEPS survey. Partnering with the George Institute for Global Health, he stressed targeting adult policies and detailed survey arms, including retail and stakeholder surveys. Mark Huffman, acknowledging National Agency for Food and Drug Administration and Control (NAFDAC) support, presented crucial findings on inadequate food labeling for low-sodium choices, advocating for improved labeling to empower consumers. Huffman proposed discussions on translating data into effective policies, including the establishment of mandatory salt limits, emphasizing the role of academic collaboration in shaping regulations. This comprehensive approach aimed to address public health concerns by fostering a more informed and health-conscious food environment.

In the context of salt reduction policies, Nicole Ide, Senior Technical Advisor, Sodium Reduction, representing g Resolve to Save Lives (RTSL), discussed pragmatic steps delineated in Table 3. These recommendations span essential components (building a data collection system, establishing reduction targets, and finally implementing, monitoring, and enforcing the plan), placing particular emphasis on the pivotal role of monitoring in shaping effective policy. RTSL, under the guidance of Nicole Ide, has strongly advocated for the imposition of mandatory sodium limits in packaged foods, deeming it the most efficacious approach for mitigating salt intake. Going beyond packaged items, RTSL has enriched the discourse by introducing two distinct models tailored to address salt reduction in non-packaged foods. Recognizing the indispensability of enforcement, RTSL underscored the imperative of establishing a comprehensive database to oversee and enforce the proposed policies. Supporting their position, RTSL, with insights from Nicole Ide, referenced the 2023 WHO global report on dietary sodium intake, underscoring the urgency and significance of the recommendations within the broader spectrum of public health [8].

**Table 3:** Steps for salt targets policy development

Steps	Description
**Step 1**: Set a goal	∙ Define country sodium intake reduction goal ∙ Use processed food contribution to intake to determine the contribution of salt targets to goal ∙ Set target date, based on feasibility and country goals ∙ Establish a monitoring schedule, to assess progress ∙ Commit to publicly reporting on changes
**Step 2**: Select Categories	A) **Comprehensive model:** All major categories of packaged food to ensure that the majority of sodium from packaged food is captured B) **Limited model:** Small number of categories that represent the foods that are major contributors of sodium to the diet
**Step 3**: Build a database	∙ Database needed to confirm draft categories, set targets, engage with industry, and monitor progress ∙ Organized by food category, containing individual product information **Key information:** - Product name and manufacturer - Unique product identifier - Nutrition information: mean/range/distribution of sodium, serving size, other nutrients of interest - Sales data (ideally but unlikely) - Updated at minimum at each target date to monitor targets
**Step 4**: Set targets	∙ Use WHO Global Sodium Benchmarks as a reference ∙ Review database and consider the mean and range of sodium within the category ∙ Understand functional properties of sodium for category (e.g., browning, moisture retention, flavour, texture) ∙ Consider product varieties within the category (e.g., plain vs whole wheat bread) ∙ Targets are usually set by mg sodium /100 g product ∙ Can set maximum targets, mean, or both ∙ If not already enacted, include requirement for back of pack nutrient declarations
**Step 5**: Implementation, monitoring and enforcement	∙ Supporting manufacturers to implement the targets ∙ Monitor progress using a database, at minimum for target years ∙ Report results publicly ∙ Use regulatory authority to penalize non-compliance ∙ Monitor changes in population salt intake over time as well
A note on engaging food industry	1) Set robust conflict-of-interest policies to guide the policy development process 2) Rely on uncompromised, independent research and evidence 3) Set a clear, transparent, and organized way for industry, partners and civil society to comment on draft categories and targets 4) Consider whether targets can be changed in response to detailed, scientific comments, and analyze data to establish the feasibility of targets

Bruce Neal and Fraser Taylor from The George Institute (TGI), Australia, shared their experiences and challenges in implementing change without mandatory regulations and working with the food industry, with a specific focus on salt reduction. They recommended crucial measures, including the implementation of mandatory salt reduction targets, extending guidelines to cover beyond health and school facilities, introducing front-of-package labeling for high-sodium foods, and establishing robust monitoring and enforcement systems. Their presentation emphasized the exploration of alternative salt formulations, including salt substitutes, that not only reduce sodium content but also incorporate potassium, a mineral linked to potential health benefits. As noted, this innovative approach, involving salt substitutes, presents a strategy for addressing excess sodium intake in the population. Delving into the development and adoption of such substitutes provides an opportunity to offer consumers healthier choices and contribute to broader public health initiatives aimed at reducing sodium-related health risks.

Joy Amafah, Global Health Advocacy Incubator (GHAI) Nigeria, In-Country Coordinator, Food and Nutrition Programs, presented GHAI’s Sodium Reduction Targets Principles—a dedicated, evidence-grounded strategy for impactful sodium reduction policies. Amafah stressed the paramount importance of transparency and the avoidance of conflicts of interest in developing sodium targets, even as GHAI acknowledges potential industry input without allowing for a decision-making role. These principles emphasize alignment with major sources of sodium consumption, existing regulatory mechanisms, and resource accessibility. GHAI’s targets aim for a feasible 20–25% reduction over 5 years at a large scale, with a progressive approach defining timelines and considerations for small and medium businesses. To address potential legal challenges, GHAI underscores the need for mandatory sodium targets based on common high-sodium food categories, utilizing a comprehensive or limited model. Effective monitoring is crucial, necessitating a database of packaged foods, back-of-pack sodium content declaration, and publicly reported company progress. GHAI further advocates for commitments to publicly report changes as integral to sodium reduction initiatives.

During the open discussion, participants actively engaged in a dialogue that encompassed several crucial topics, including the potential unintended consequences of dietary policies (such as cultural and social implications; dietary policies may not always align with cultural or social norms, leading to resistance or unintended social consequences), the utilization of iodine supplements, the role of the food industry in sodium reduction efforts, and the range of strategies to mitigate excess dietary sodium intake. The discourse reflected a shared consensus among stakeholders regarding the imperative to intensify actions beyond the current provisions in the NMSAP. The acknowledgement of the need for heightened efforts suggests a collective understanding of the evolving challenges and complexities in the realm of dietary health. This consensus underscores the commitment of stakeholders to address emerging issues, fostering a proactive approach in aligning policies with the dynamic landscape of public health concerns.

The meeting concluded with remarks from Nigerian government agencies and organizations. They discussed specific actions which included rigorous food system monitoring from the national level by relevant government agencies such as NAFDAC, front-of-package labeling to help consumers make healthier purchases, defining and implementing mandatory sodium limits for packaged and unpackaged food products and school meals, implementing educational campaigns spanning diverse sectors on reducing salt use, raising awareness through mass mobilization campaigns, and advocating for salt reduction in fast food outlets. Participants also recommended robust partnerships with health organizations and the empowerment of nutrition educators to foster healthier food environments and dietary choices of consumers. In addition to the key actions outlined, several noteworthy points were raised during the discussion. One crucial aspect highlighted was the existing funding challenges, indicating the need for financial support and resources to effectively implement the proposed strategies. Moreover, the significance of training nutrition educators was underscored, emphasizing the pivotal role they play in disseminating accurate information and promoting healthier dietary choices among the public. Lastly, the potential of low-sodium, potassium-enriched salt substitutes was explored, suggesting a possible avenue for innovation and alternative solutions to address excess sodium intake in the population. These considerations further contribute to the comprehensive approach needed to tackle dietary sodium reduction in Nigeria.

## Conclusions and recommendations

The recommendations arising from the NaSS policymakers’ meeting encompass a comprehensive approach to combating excessive dietary sodium intake in Nigeria. These recommendations first include developing evidence-based policies, including strengthening existing policies through collaborative efforts across sectors and strengthening surveillance systems to monitor sodium consumption patterns and assess policy impact. Second, the engagement of industries was encouraged to reformulate products, reduce sodium content, and improve product labeling for informed consumer choices. Third, there was an emphasis on public engagement to positively influence policy outcomes and raise awareness about the risk of high sodium consumption. Fourth, creating stringent monitoring mechanisms, including mandatory sodium limits, was advised for both packaged and non-packaged foods. Fifth, educational campaigns spanning various sectors, collaboration with health organizations, front-of-package labeling, and training of nutrition educators were encouraged to foster healthier food environments and dietary choices. Sixth, the exploration of low-sodium, potassium-enriched salt substitutes were recommended to reduce sodium intake associated with discretionary salt intake. Lastly, a multi-sectoral approach involving government agencies, the food industry, non-governmental organizations, academia, and international partners to address the challenge of reducing excess dietary sodium consumption collectively was highly advocated (Table 4).

**Table 4:** Next steps / priority actions: multi-sectoral approach and priority recommendations from the NaSS policymakers’ meeting on reducing excess dietary sodium consumption in Nigeria

Monitoring and Enforcement	∙ Establish robust monitoring systems. ∙ Develop a comprehensive database to track progress. ∙ Enforce regulations effectively. ∙ Measure the impact of regulatory interventions.
Mandatory Sodium Limits	∙ Consider adopting mandatory sodium limits for packaged foods. ∙ Explore extending limits to non-packaged foods. ∙ Learn from successful implementation in other countries.
Front-of-Package Labeling	∙ Explore the implementation of front-of-package labeling. ∙ Focus on high-sodium foods. ∙ Empower consumers with clear information. ∙ Consider experiences and recommendations from successful initiatives, such as in Australia and the United Kingdom.
Public Engagement	∙ Recognize the importance of engaging various stakeholders. ∙ Develop strategies for knowledge dissemination. ∙ Conduct public awareness campaigns. ∙ Involve communities to garner support.
Cultural Sensitivity	∙ Acknowledge potential unintended consequences. ∙ Consider cultural and social implications. ∙ Ensure policies align with cultural norms. ∙ Mitigate resistance and unintended social consequences.
International Collaboration	∙ Leverage global experiences and collaborations. ∙ Refer to insights from international organizations. ∙ Enrich local strategies. ∙ Align with global best practices.

## Data Availability

This study was conducted with the active participation and collaboration of relevant stakeholders, during which detailed notes were taken to document discussions and decisions. The notes generated during the meetings are available from the corresponding author upon request.
